# Finger Gesture Recognition Using Sensing and Classification of Surface Electromyography Signals With High-Precision Wireless Surface Electromyography Sensors

**DOI:** 10.3389/fncom.2021.770692

**Published:** 2021-11-11

**Authors:** Jianting Fu, Shizhou Cao, Linqin Cai, Lechan Yang

**Affiliations:** ^1^Chongqing Institute of Green and Intelligent Technology, Chinese Academy of Sciences, Chongqing, China; ^2^School of Automation, Chongqing University of Posts and Telecommunications, Chongqing, China; ^3^Department of Soft Engineering, Jinling Institute of Technology, Nanjing, China

**Keywords:** surface EMG, EMG sensor, finger gesture recognition, convolution neural network, artificial limb

## Abstract

Finger gesture recognition (FGR) plays a crucial role in achieving, for example, artificial limb control and human-computer interaction. Currently, the most common methods of FGR are visual-based, voice-based, and surface electromyography (EMG)-based ones. Among them, surface EMG-based FGR is very popular and successful because surface EMG is a cumulative bioelectric signal from the surface of the skin that can accurately and intuitively represent the force of the fingers. However, existing surface EMG-based methods still cannot fully satisfy the required recognition accuracy for artificial limb control as the lack of high-precision sensor and high-accurate recognition model. To address this issue, this study proposes a novel FGR model that consists of sensing and classification of surface EMG signals (SC-FGR). In the proposed SC-FGR model, wireless sensors with high-precision surface EMG are first developed for acquiring multichannel surface EMG signals from the forearm. Its resolution is 16 Bits, the sampling rate is 2 kHz, the common-mode rejection ratio (CMRR) is less than 70 dB, and the short-circuit noise (SCN) is less than 1.5 μV. In addition, a convolution neural network (CNN)-based classification algorithm is proposed to achieve FGR based on acquired surface EMG signals. The CNN is trained on a spectrum map transformed from the time-domain surface EMG by continuous wavelet transform (CWT). To evaluate the proposed SC-FGR model, we compared it with seven state-of-the-art models. The experimental results demonstrate that SC-FGR achieves 97.5% recognition accuracy on eight kinds of finger gestures with five subjects, which is much higher than that of comparable models.

## Introduction

Comparing to traditional peripheral devices such as a mouse or a keyboard, finger gesture recognition (FGR) is much more convenient and natural for users to control an artificial limb and to interact with a computer (Rechy-Ramirez and Hu, 2015). As a result, FGR becomes more and more important during the past few years (Rechy-Ramirez and Hu, 2015). Currently, the most common methods of FGR are visual-based, voice-based, and surface electromyography (EMG)-based ones. Among them, surface EMG is the comprehensive photoelectrical signal of potential muscle action on the surface of the skin (Botros et al., 2020). It is a kind of non-stationary signal, and its strength is sensitively proportional to the degree of muscle activity, which makes it can accurately represent the gesture of fingers (Botros et al., 2020). Therefore, surface EMG-based is widely adopted to achieve FGR.

Surface EMG-based FGR has been researched for many years. Among existing approaches, machine learning-based approach is very popular and successful (Qi et al., 2020; Wong et al., 2021). For example, Phinyomark et al. (2011) applied the critical index analysis and fractal dimension to extract the characteristics of surface EMG signals, and seven kinds of gestures were recognized from eight-channel EMG signals. Ishii et al. (2012) divided hand motions into six movements and classified finger motions using two types of characteristics. Khushaba et al. (2016) proposed the mutual component analysis (MCA) by improving the principal component analysis (PCA) to deduct the noise and redundant features. The recognition accuracy reached 95% for 15 kinds of gestures by combining the feature selection and MCA from eight channels of the surface EMG signals. Ngeo et al. (2014) used the multi-output convolution Gaussian process to analyze the dependence of multi-joint gesture and to estimate the finger joint motion. Through the correlation between knuckles, the regression model was modified to improve the recognition rate of finger posture. AlOmari and Liu (2015) constructed a model by combining genetic algorithm, particle swarm optimization, and support vector machine (SVM). Arozi et al. (2020) identified the hand gesture through the single channel of the surface EMG signal with the time-domain feature extraction, PCA, feature dimensionality reduction, and neural network. The recognition accuracy is 86.7% for nine kinds of gestures.

Recently, since convolution neural network (CNN) was proposed by Krizhevsky et al. in 2012 (Atzori et al., 2016), it has achieved great success in many fields of image recognition, natural language processing, and language translation (Wu et al., 2019b; Yao et al., 2019). As it has much better performance of feature extraction and non-linear fitting than traditional machine learning models, many researchers employed CNN to classify hand gestures from surface EMG signals. For example, Atzori et al. (2016) and Geng et al. (2016) selected CNN to classify hand gestures using the original surface EMG signals as the input signal. A spectral map that was obtained by the short-time Fourier transform (STFT) from the original surface EMG signal was put into the convolution network (Du et al., 2017; Côté-Allard et al., 2019a). Zia Ur Rehman et al. (2018) constructed a simple network model consisting of one convolutional layer, one pooling layer, and two fully connected layers. Then, the original surface EMG was directly used as the input of the CNN. Wu et al. (2018) proposed a model based on long short-term memory (LSTM) and CNN, where LSTM reserves time information and CNN extract features. Its performance was better than the model proposed in the study by Santello et al. (2016). Chen L. et al. (2020) designed a compact CNN with a small number of parameters to improve the classification accuracy of EMG signals. However, all these approaches mainly focus on developing a CNN-based recognition model while ignoring to acquire the high-precision surface EMG. Hence, they still cannot fully satisfy the required recognition accuracy for real applications of artificial limb control and human-computer interaction.

**FIGURE 1:**
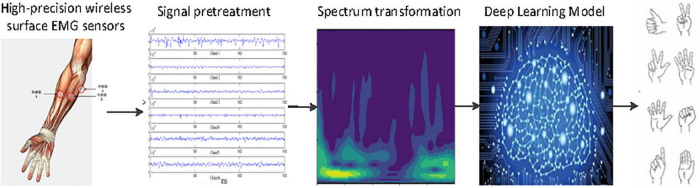
General chart of finger gesture recognition (FGR) using sensing and classification of surface electromyography (EMG) signals (SC-FGR).

To address this issue, this study proposes a novel FGR model that consists of two parts, namely, sensing and classification of surface EMG signal (SC-FGR). First, wireless sensors with high-precision surface EMG are developed for acquiring multichannel surface EMG signals from the forearm. Second, a CNN-based classification algorithm is proposed to classify the acquired surface EMG signals for FGR, where we named it CNN-FGR. A general chart of FGR with the proposed SC-FGR model is shown in Figure 1. The surface EMG signals of each channel are segmented by a moving window. A spectrum map is generated by continuous wavelet transform (CWT) from the segmented signals of each channel. Then, the spectrum maps of multiple channels are put into the CNN-FGR for classifying.

The main research contents and contributions of this study are as follows:

(1) The wireless sensors are specially developed to acquire surface EMG from the forearm with high precision. Its resolution is 16 Bits, the sampling rate is 2 kHz, the common-mode rejection ratio (CMRR) is less than 70 dB, and the short-circuit noise (SCN) is less than 1.5 μV.
(2) A new CNN-FGR algorithm is proposed to accurately classify the surface EMG signals acquired by the developed wireless sensors. It consists of a 5-layer CNN that is trained on a spectrum map transformed from the time-domain signals of surface EMG by CWT.
(3) A novel SC-FGR model is proposed for highly accurate FGR. It comprises two parts of the developed wireless sensors and the proposed CNN-FGR algorithm.
(4) A surface EMG dataset is collected and shared online. It contains eight kinds of finger gestures with five subjects collected by the developed wireless sensors.

In the experiments, we evaluated the proposed SC-FGR model on the collected surface EMG dataset. The results demonstrate that the proposed SC-FGR model achieves 97.5% recognition accuracy, which is much higher than that of comparable models.

The rest of this article is organized as follows: A wireless surface EMG acquisition system is designed in section “A Wireless Surface EMG Acquisition System”; The data processing and CNN-FGR algorithm are described in detail in section “Data Processing and Network Architecture”; The proposed SC-FGR model is compared with several related models in Section “Experiment and Results”; and finally, section “Conclusion” concludes this study.

## A Wireless Surface EMG Acquisition System

The EMG is a weak electrophysiological signal of a muscle fiber group. It can be detected by sensors placed on the surface of skin or needle sensors implanted in muscle tissue (De Luca et al., 2006). The EMG signal is closely related to neuron muscular activity information so that the surface EMG signals of the forearm can be used to analyze and recognize the finger gestures.

**FIGURE 2:**
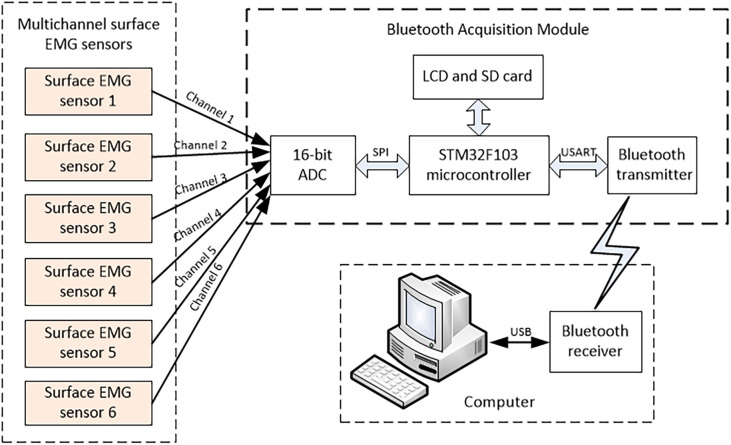
Multichannel surface EMG acquisition.

De Luca (1997) showed that the amplitude of the EMG signal was random and could be expressed by the arithmetic mean value of zero Gaussian distribution function. The surface EMG signal is a weak signal whose amplitude ranges from 0 to 10 mV (Peak-to-Peak) or 0 to 1.5 mV [root mean square (RMS)]. The frequency range of the available energy signal is limited from 0 to 1,000 Hz, and the dominant energy is distributed in the range from 50 to 150 Hz. In the same state of muscle motion, the amplitude-frequency characteristic curve of the EMG signal is similar, and the EMG signal has a certain regularity in the muscle motion state of different detection points. According to the characteristics of surface EMG, the frame of the acquisition module is designed as shown in Figure 2.

Inspired by the surface EMG sensor on the market, the surface EMG sensor consists of the surface EMG electrode and the signal conditioning circuit. This surface EMG sensor uses three parallel silver electrodes with a spacing of 10 mm, including two measuring electrodes and one reference electrode, which prevent saturation caused by the common-mode signals. The silver electrode is put close to the skin for complete polarization, forming a capacitor by surface skin and electrode. To improve the accuracy, the front analog amplifier circuit is designed as close as possible to the silver electrode. This measure is beneficial to weaken the disturbance of white noise for the acquisition of surface EMG signals. Then, the potential difference between the two measuring electrodes is detected by the differential amplifier circuit and converted into a digital signal for signal preprocessing. Finally, the digital signal is transformed into a computer by the Bluetooth data acquisition module.

**FIGURE 3:**
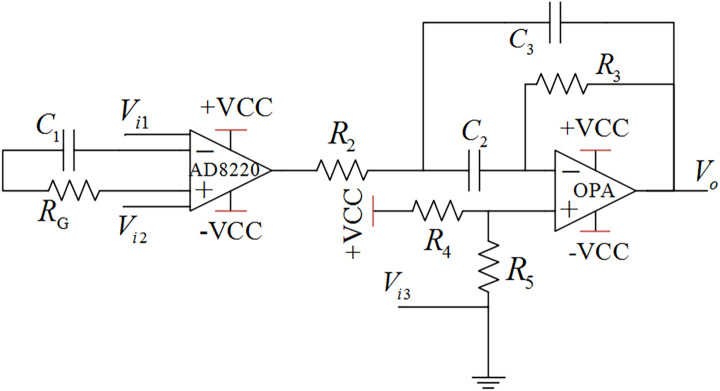
Conditioning circuit for the analog signal.

**FIGURE 4:**
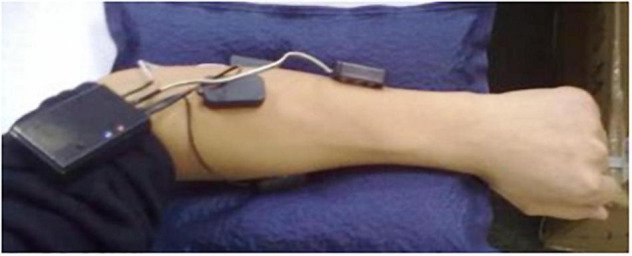
Multichannel wireless surface EMG acquisition device.

The signal conditioning circuit plays a key role in amplifying the weak signal to improve the performance of the whole acquisition system. The expected conditioning circuit is with high input impedance, high gain, wide frequency band, low noise, and high CMRR. It should amplify surface EMG signals while suppressing other noise signals (Khokhar et al., 2010). The signal conditioning circuit uses instrument amplifier AD8220 with the JFET as the input of the preamplifier. The rail-to-rail amplifier OPA364 constitutes the band-pass amplifier. The instrument amplifier AD8220 plays the role of first-order high-pass filtering, while the amplifier OPA364 plays the role of second-order band-pass filtering. All in all, the function of the analog conditioning circuit is to amplify the original EMG signal 1,000 times and then signal processing by the second-order band-pass filtering with the range of 5–1,000 Hz. The schematic diagram of the signal conditioning circuit (Fu et al., 2013) is shown in Figure 3. The theoretical gain of the signal conditioning circuit is shown as follows:


(1) $$\text{G}=\frac{V_{o}}{V_{i\,2}-V_{i\,1}}$$

$$=\left(\frac{49.4\,e^{3}}{R_{G}+R_{c_{1}}}+1\right)\,\left(\frac{R_{3}\,R_{c_{3}}}{R_{c_{2}}\,R_{c_{3}}+R_{2}\,R_{c_{2}}+R_{2}\,R_{c_{3}}+R_{2}\,R_{3}}\right)$$

where G represents amplifier gain; R_*c*1_,R_*c*2_, and R_*c*3_ represent the impedance of the capacitance C_1_,C_2_, and C_3_, respectively;V_*i*1_,V_*i*2_, and V_*i*3_represent the input of the detection points; and V_*o*_ is the output of the signal conditioning circuit. The core design principles of the surface EMG acquisition system are anti-noise treatment, such as co-ground and anti-electromagnetic interference. This EMG acquisition system uses a Bluetooth module for physical isolation and anti-interference, avoiding 50-Hz interference from a wired connection with the computer. This data acquisition system contains a 16-bit AD conversion, an ARM processor, and a Bluetooth communication module, as shown in Figure 2. The output of the surface EMG sensor is connected to the input port of the AD converter by shielding line. It adopts the common ground technology between the analog signal and the digital signal. There is photoelectric isolation between the AD converter and the ARM microprocessor to reduce the crosstalk from digital signals to analog signals. On the one hand, the ARM controller stores the eigenvalues of the collected signal and stresses it in the local SD card. On the other hand, it transfers the collected signal to the HC-05 Bluetooth module through the USRT serial communication protocol. Bluetooth communication realizes the information interaction function between sensors and the computer. The Bluetooth communication module uses low-energy radio communication technology to realize data transmission, with the maximum rate of 1 Mb/s (Song et al., 2020) and the effective communication of 15 m. The multichannel wireless surface EMG module is designed with a highly extending function and could be extended to 4–8 channels. The surface EMG device is shown in Figure 4.

The parameter comparison between the high-precision wireless surface EMG acquisition system and the other surface EMG acquisition systems on the market is shown in Table 1.

**TABLE 1:** Characterization of different surface electromyography (EMG) acquisition systems.

	**Delsys Trigno Wireless EMG** (ADInstituments, 2020)	**Biometrics DataLITE sEMG** (Côté-Allard et al., 2019b)	**Thalmic Labs MYO Armhand** (Côté-Allard et al., 2019b)	**This design**
Number of channels	16	16	8	**4–8**
sEMG ADC	16 bits	13 bits	8 bits	**16 bits**
Sampling rate	2,000 Hz	2,000 Hz	1,000 Hz	**2,000 Hz**
Bandwidth	10–850 Hz	10–490 Hz	5–100 Hz	**5–1,000 Hz**
Contact material	Sliver	Stainless Steel	Stainless Steel	**Sliver**
Common-mode rejection ratio	>80 dB	N.A	N.A	**>70 dB**
Short-circuit noise	<0.75 μV	<5 μV	N.A	** < 1.5 μV**
Transfer protocol	BLE 4.2	WiFi	BLE 4.0	**BLE 4.2**

## Data Processing and Network Architecture

### Signal Feature Extraction of Surface EMG

Since the surface EMG signal is non-stationary, it is limited to analysis the signal with Fourier transform. The STFT, which divides the signal into smaller segments by sliding windows and calculates the Fourier transform of each segment separately, is an effective method to solve that problem. A frequency spectrogram can be obtained from the transformation of STFT. When the signal *x*(*t*) and window function *w*(*t*) are designed, the spectra can be calculated as follows:


(2) $$s\,p\,e\,c\,t\,r\,o\,g\,r\,a\,m\,\left(x\,\left(t\right),w\,\left(t\right)\right)={\left|S\,T\,F\,T_{x}\,\left(t,f\right)\right|}^{2}$$

(3) $$S\,T\,F\,T_{x}\,\left(t,f\right)=\int_{-\infty }^{+\infty }\left[x\,\left(u\right)\,w\,\left(u-t\right)\right]\,e^{-j\,2\,\pi \,f\,u}\,du$$

where *f*represents the frequency. The wavelet transform (WT) is similar to STFT, while it overcomes the disadvantage that the window does not change with frequency in STFT. By adjusting the width of the window, the WT adapts to the frequency changes in the signal. When the frequency of the processed signal increases, the WT improves the resolution by narrowing the time window. Furthermore, WT is an ideal analysis tool, which can obtain the amplitude and frequency of mutations in the signal.


(4) $$X\,\left(a,b\right)=\frac{1}{\sqrt{b}}\,\int_{-\infty }^{\infty }x\,\left(t\right)\,\phi \,\left(\frac{t-a}{b}\right)\,dt$$

(5) $$\int_{-\infty }^{+\infty }\frac{{\left|\phi \,\left(\omega \right)\right|}^{2}}{\omega }\,d\omega <\infty$$

where the Fourier transform φ(*w*) must satisfy Equation 5. φ(*t*)is named as the parent wavelet function, which is a signal with limited duration, frequency change, and zero mean value. The scaling factor *b* and the translation factor *a* control the scaling and transform of the wavelet function, respectively. There are many kinds of parent wavelet functions for the transform, such as Mexican hat wavelet (MEXH), Gaussian wavelet (GAUS), complex Morlet wavelet (CMOR), Shannon wavelet (SHAN), frequency B-spline wavelet (FBSP), and complex Gaussian wavelet (CGAU). MEXH function is defined by Equation 6 as follows:


(6) $$\psi \,\left(t\right)=c\,\left(1-t^{2}\right)\,e^{-t^{2}/2}$$

where $c=\frac{2}{\sqrt{3}}\,\pi^{1/4}$. GAUS is the differential form derived from the Gaussian function. It is defined by Equation 7 as follows:


(7) $$\psi \,\left(t\right)=C_{p\,1}\,t\,e^{-t^{2}}$$

where $C_{p1}=\sqrt[{4}]{2/\text{π}}$. CMOR is defined by Equation 8 in the time-domain and by Equation 9 in the frequency domain as follows:


(8) $$\psi \,\left(t\right)=\frac{1}{\sqrt{\pi \,f_{b}}}∙e^{j\,2\,\pi \,f_{c}\,t-\left(t^{2}/f_{b}\right)}$$

(9) $$\Psi \,\left(f\right)=e^{\pi^{2}\,f_{b}\,{\left(f-f_{c}\right)}^{2}}$$

where *f*_*c*_ is the center frequency and *f*_*b*_ is the bandwidth. SHAN is defined by Equation 10 as follows:


(10) $$\psi \,\left(t\right)=\sqrt{f_{b}}\,\sin c\,\left(f_{b}\,x\right)\,e^{2\,i\,\pi \,f_{c}\,x}$$

where *f*_*c*_ is the center frequency and *f*_*b*_ is the bandwidth. FBSP is defined by Equation 11 as follows:


(11) $$\psi \,\left(t\right)=\sqrt{f_{b}}\,{\left[\sin\left(\frac{f_{b}\,t}{m}\right)\right]}^{m}\,e^{2\,j\,\pi \,f_{c}\,t}$$

where *m* is an integer parameter, *f*_*c*_ is the center frequency, and *f*_*b*_ is the bandwidth. CGAU is defined by Equation 12 as follows:


(12) $$\psi \,\left(t\right)=C_{p}\,e^{-i\,t}\,e^{-x^{2}}$$

where *C*_*p*_ is constant.

**FIGURE 5:**
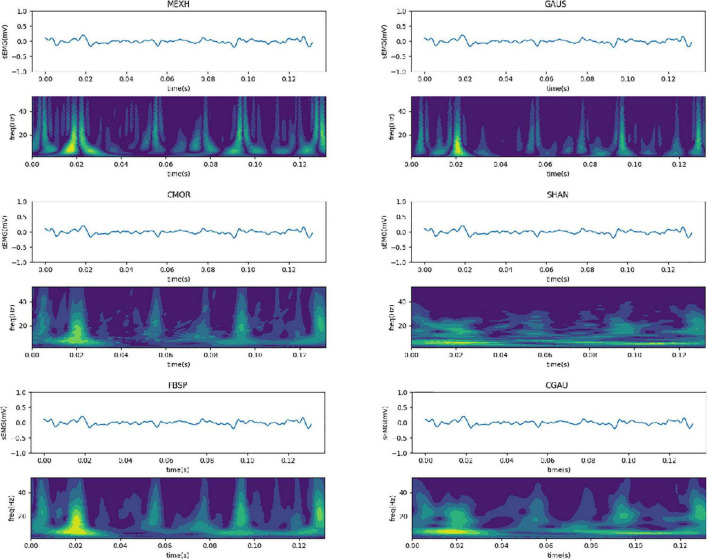
Spectrum maps transformed from surface EMG with different kinds of parent wavelet functions.

After the CWT of the surface EMG signals, the corresponding spectrum map is similar to the image on the scale and also contains the frequency domain information of the timing sequence data. The six-channel surface EMG signals of the forearm were collected by the high-precision wireless surface EMG sensors, and the data of each channel were separated by applying a sliding window of 264 samples (132 ms). The parent wavelet of the CWT adopts the optimal wavelet function, calculating the CWTs with 64 scales to obtain the 64 × 264 matrix of spectral information. The matrix is set as input to the CNN-FGR algorithm. Thus, the input of the CNN-FGR algorithm has six channels, each consisting of a matrix with the size of 64 × 264. Figure 5 is the spectrum maps of the spectral information transformed from 264 EMG data with different kinds of parent wavelet functions, such as MEXH, GAUS, CMOR, SHAN, FBSP, and CGAU.

### CNN-FGR Algorithm

**FIGURE 6:**
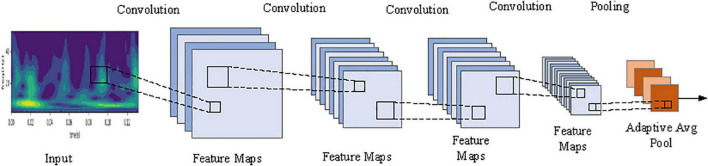
The block diagram of the CNN-FGR algorithm.

Chen L. et al. (2020) used a compact CNN to improve the hand gesture recognition by surface EMG. Inspired from that model, the CNN-FGR algorithm consists of four convolutional layers and one mean pool layer as shown in Figure 6, and its design details are listed in Table 2.

**TABLE 2:** Configuration of CNN of CNN-FGR algorithm.

**Layers of Network**	**Parameters of each layer**
Convolutional layer 1 (Activation Function: ReLU)	kernel_size = 3, stride = 1 Number of feature graphs:16
Convolutional layer 2 (Activation Function: ReLU)	kernel_size = 3, stride = 2 Number of feature graphs:32
Dropout	*P* = 0.5
Convolutional layer 3 (Activation Function: ReLU)	kernel_size = 3, stride = 1 Number of feature graphs:32
Convolutional layer 4 (Activation Function: ReLU)	kernel_size = 3, stride = 2 Number of feature graphs: 64
Convolutional layer 5 (Activation Function: ReLU)	adaptive_avg_pool2d

The loss function is calculated as follows:


(13) $$L\,o\,s\,s=-\sum_{i=1}^{n}y_{i}\,\log\left(y_{i}^{\prime }\right)$$

where *y*_*i*_ is the true value of the first class, *n* is the number of categories, *y*_*i*_′ is the first-class prediction value of the output. Since one-hot coding was adopted, the true value of one class is 1, while the true value of the other classes is 0.

The three quantities where accuracy rate (AR) is used to evaluate the performance of the SC-FGR model, such as AR, the mean AR (MAR), and the SD of AR (SD-AR), are, respectively, computed as Equations 14–16. A test set composed of *t* number of instances *x*_*i*_ with ω known is used for the test stage.


(14) $$A\,R=\frac{1}{t}\,\sum_{i=1}^{t}\Psi \,\left(w,f\,\left(x_{i}\right)\right),\Psi \,\left(w,f\,\left(x_{i}\right)\right)$$

$$=\left\{ \begin{array}{cc} 1,i\,f\,w=f\,\left(x_{i}\right) & \\ 0,e\,l\,s\,e & \end{array}\right.$$

(15) $$M\,A\,R=\frac{1}{n}\,\sum_{k=1}^{n}A\,R_{k}$$

(16) $$S\,D-A\,R=\sqrt{\frac{1}{n}\,\sum_{k=1}^{n}{\left(A\,R_{k}-M\,A\,R\right)}^{2}}$$

where *f*(*x*_*i*_) represents the calculated label of *x*_*i*_, and *n* is the repeated times of computing AR. MAR represents the classification ability of the algorithm, and SD-AR represents the robustness of the algorithm.

Advanced optimization methods were used for the backpropagation of the CNN-FGR algorithm with the ultimate goal to minimize the function loss. In the field of image recognition, the common size of the convolutional kernel is selected as 3 × 3, 5 × 5, or 7 × 7 (Krizhevsky et al., 2012; Simonyan and Zisserman, 2014). Therefore, the different sizes of the convolutional kernel in the CNN-FGR algorithm model are evaluated to get a better experimental result. Meanwhile, the various layer feature maps of the model are also set smaller to minimize the parameters of the model. The step length of the convolution is set to 2, for reducing the feature parameters by half. To further reduce the number of network parameters, the output of the model used the convolutional layer with adaptive mean sampling for classification, instead of the full connection layer.

## Experiment and Results

### Finger Gestures

**FIGURE 7:**
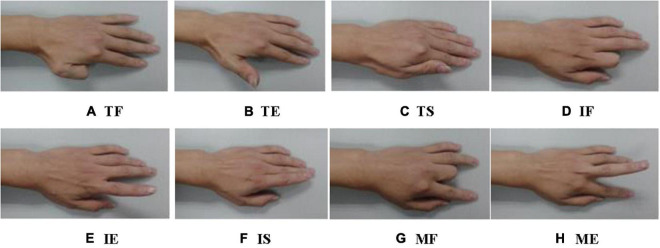
Eight kinds of finger gestures: **(A)** Thumb Flection (TF), **(B)** Thumb Extension (TE), **(C)** Thumb Swing (TS), **(D)** Index-finger Flection (IF), **(E)** Index-finger Extension (IE), **(F)** Index-finger Swing (IS), **(G)** Middle-finger Flection (MF), and **(H)** Middle-finger Extension (ME).

Before the experiment, the collection points of the surface EMG from the forearm must be disinfected and cleaned to reduce skin contact interference. In the experiment, the subject sat on a chair with his left arm lying flat on the table and relaxed. In each group of experiments, as shown in Figure 7, each subject completed eight types of gestures, namely, Thumb Flection (TF), Thumb Extension (TE), Thumb Swing (TS), Index-finger Flection (IF), Index-finger Extension (IE), Index-finger Swing (IS), Middle-finger Flection (MF), and Middle-finger Extension (ME).

### Number of Sensors and Layout of Detection Points

**FIGURE 8:**
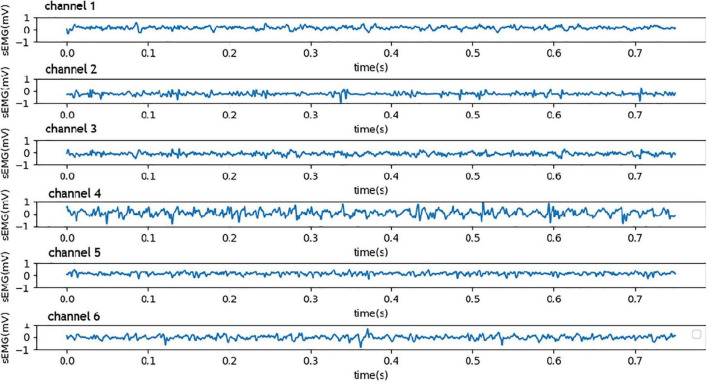
Six channels of the raw EMG signals from the high-precision sensors.

The surface EMG signal is closely related not only to the objective factors such as human physical state and movement state but also to the form and location of the detection electrode. The number of electrodes also has a great impact on the accuracy of surface EMG signal recognition. Extensive research and experiments showed that the acquisition of surface EMG signals with six channels can not only effectively identify single and multi-finger movement information but also avoid the waste of resources with over-channel detection. It was found that the electrodes were placed on the nerve-dominated region, and the EMG signals collected in the 10-tendon head or muscle edge area were usually weak. When sensors were placed vertically on the muscle fibers, the surface EMG signals were strongest. Since the front group muscles of the forearm cover the flexor, it mainly controls the bending movement of the elbow, wrist, and knuckles. The muscles of the back group cover the stretched muscles, which mainly control the stretching movement of each joint. In this experiment, six surface electrodes were placed on the corresponding muscle abs, and the electrodes were radially perpendicular to the muscle fibers. The sensors were fixed on the forearm with a bandage in moderate tension. Three sensors were placed on the corresponding muscle abs at the front of the forearm, mainly for detecting the bending movement of the finger, while the other sensors were placed at the back of the forearm for detecting the stretching movement of the fingers. The raw EMG signals detected by six sensors on the forearm are shown in Figure 8.

### Classification Results

This experiment used the high-precision wireless surface EMG sensors and DELSYS data acquisition system to collect six channels of the surface EMG signal, with a frequency of 2 kHz. Before classification, the collected surface EMG signal must be pretreated and feature extracted. The original EMG signal is preprocessed with a 264-sample-point (132 ms) sliding window and a 100-sample-point incremental step. After the data segment processing, each experiment of each gesture obtains 12 samples, and 300 samples are collated after 25 repeating times. The total datasets of eight gestures of five subjects (i.e., S1, S2, S3, S4, and S5) are 12,000 samples. Each subject has 2,400 samples, where 1,920 samples are adopted as training set and 480 samples are adopted as testing set.

To evaluate the effects of CWT in transforming the surface EMG from time-domain to spectrum map, we, respectively, trained the CNN-FGR algorithm on the time-domain and the spectrum map of surface EMG. The comparison results on the testing set are shown in Table 3, where we observed that the CNN-FGR algorithm trained on the spectrum map of surface EMG achieves much higher accuracy than that trained on the time-domain of surface EMG. This observation demonstrates that transforming the surface EMG from time-domain to spectrum map by CWT is beneficial for the CNN-FGR algorithm to achieve a better performance of FGR.

**TABLE 3:** The effects of continuous wavelet transform (CWT) on the accuracy of gesture classification of five subjects (S1, S2, S3, S4, and S5).

	**S1**	**S2**	**S3**	**S4**	**S5**	**MAR**	**SD-AR**
Time-domain (%)	92.3	94.37	97.5	95.4	98.54	95.62	2.22
Spectrum map (%)	92.50	97.50	97.50	100.00	100.00	97.50	2.74

There are two factors affecting the identification accuracy in the SC-FGR algorithm model. One is the size of the convolutional kernel, and the other is the parent wavelet function. Using the same parent wavelet function “CGAU” for CWT transform, the different sizes of the convolutional kernel are compared to get a better recognition accuracy. The training accuracy curve, loss curve during training, and testing accuracy curve are used to analyze the results of FGR. The accuracy of the CNN-FGR algorithm with the convolutional kernel size of 3 × 3 is shown in Table 4.

**TABLE 4:** The accuracy of the CNN-FGR algorithm with the convolutional kernel size of 3 × 3 on five subjects.

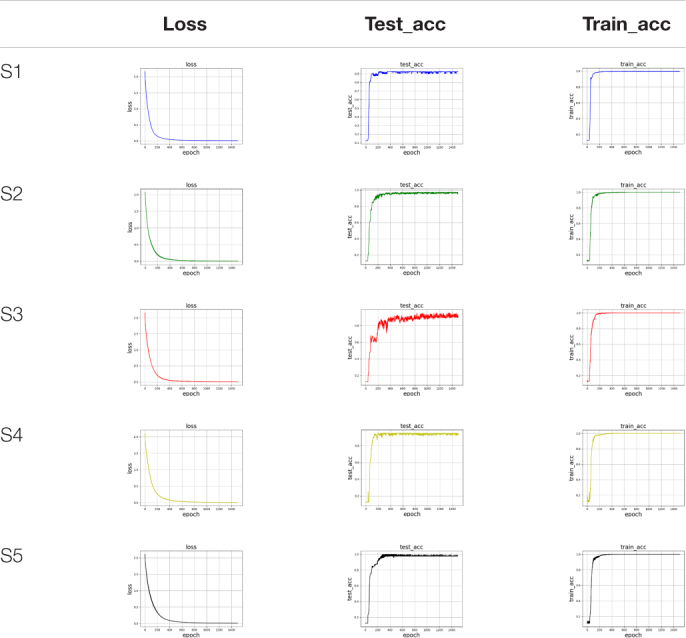

From Table 4, we found that the training accuracy keeps increasing and loss keeps decreasing with more epochs until reaching convergence. Similarly, testing accuracy also keeps increasing with more epochs until reaching convergence. These findings verify that the CNN-FGR algorithm can be well applied to classify these samples for FGR. In the experiment, we compared the accuracy of the CNN-FGR algorithm with the kernel size of 3 × 3, 5 × 5, 7 × 7, and 9 × 9 on collected datasets.

From Table 5, it can be observed that the classification ability of the algorithm is improved, but the robustness of the algorithm becomes worse, while the size of the convolution kernel increases. The size of 5 × 5 is a better selection as the convolution kernel, because not only the accuracy is high, but also the robustness performed well.

**TABLE 5:** The comparison of accuracy with different convolution kernel sizes.

	**S1**	**S2**	**S3**	**S4**	**S5**	**MAR**	**SD-AR**
3 × 3 (%)	92.5	97.5	97.5	96.67	100	96.83	2.44
5 × 5 (%)	**92.5**	**97.5**	**96.875**	**98.58**	**100**	**97.09**	**2.53**
7 × 7 (%)	92.5	100	97.5	97.29	100	97.46	2.74
9 × 9 (%)	92.5	100	97.71	98.125	100	97.67	2.75

To choose the suitable parent wavelet function for CNN-FGR, the experiments are carried out on different parent wavelet functions, such as MEXH, SHAN, GAUS, FBSP, CGAU, and CMOR. For dataset S3, the comparison results of accuracy of various parent wavelet functions with the same convolutional kernel size of 5 × 5 are shown in Table 6.

**TABLE 6:** The comparison results of accuracy of various parent wavelet functions on the dataset S3.

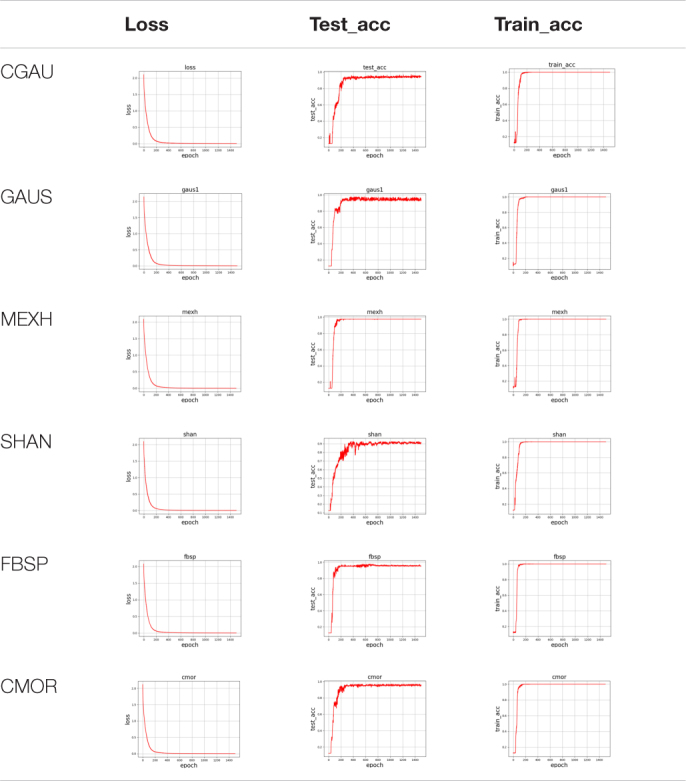

On all collected datasets, the comparison results of accuracy of various parent wavelet functions with the same convolutional kernel size of 5 × 5 are shown in Table 7.

**TABLE 7:** The comparison results of accuracy of various parent wavelet functions on the collected datasets.

	**S1**	**S2**	**S3**	**S4**	**S5**	**MAR**	**SD-AR**
MEXH (%)	92.50	98.75	97.50	97.71	98.75	97.04	2.33
SHAN (%)	92.50	98.54	92.50	98.38	96.67	95.72	2.71
FBSP (%)	92.50	98.13	97.91	96.67	100.00	97.04	2.51
CMOR (%)	92.50	98.33	97.50	97.5	100.00	97.17	2.51
CGAU (%)	92.50	97.50	96.88	98.54	100.00	97.08	2.52
GAUS (%)	**92.50**	**97.50**	**97.50**	**100.00**	**100.00**	**97.50**	**2.74**

From Table 7, it is easy to get the results that the accuracy of GAUS is higher than that of other wavelet functions, but the robustness is worse. Considering the classification ability and the robustness, the algorithm with the parent wavelet MEXH performs better.

Finally, to evaluate the proposed SC-FGR model, we compared it with several related models. Especially, enhanced time-domain (EnhancedTD) (Khushaba et al., 2016; Fournelle and Bost, 2019), time-domain cycle (TDC) (Tang et al., 2010), autoregression (AR) (Soares et al., 2003), sample entropy (SampEn) (Delgado-Bonal and Marshak, 2019), and wavelet package coefficient (WPC) (Zhao et al., 2006) are selected as feature extractors. The classical classifiers [e.g., probabilistic neural network (PNN) (Zeinali and Story, 2017), linear discriminant analysis (LDA) (Zhang et al., 2012), and SVM (Varatharajan et al., 2018)], CNN (Chen H.F. et al., 2020), and CWT-EMGNet (Chen L. et al., 2020) are adopted as classifiers. The comparison results are recorded in Table 8, where we clearly observed that the SC-FGR model achieves 97.5% accuracy, which is the best among all the models. Hence, we concluded that the proposed SC-FGR model is powerful for FGR.

**TABLE 8:** The comparison results of accuracy of various models on the collected datasets.

	**S1**	**S2**	**S3**	**S4**	**S5**	**MAR**	**SD-AR**
TDC-AR+PCA+PNN (%) (Fu et al., 2017)	90.84	89.39	93.88	95.8	95.7	93.12	2.59
TDC-WPC+PCA+PNN (%)	90.67	91.45	95.78	98.17	97.54	94.72	3.10
EnhancedTD+LDA (%) (Zhang et al., 2012)	89.58	92.41	94.13	95.81	97.48	93.88	2.73
EnhancedTD+SVM (%)	88.29	90.57	94.06	93.59	95.03	92.31	2.50
SampEn+LDA (%)	89.67	92.75	96.22	95.77	94.07	93.70	2.36
SampEn+SVM (%)	87.44	90.77	93.18	94.44	93.82	91.93	2.57
CNN (%) (Chen H.F. et al., 2020)	92.3	94.37	97.5	95.4	98.54	95.62	2.22
CWT+EMGNet (%) (Chen L. et al., 2020)	92.5	94.58	96.875	99.17	99.58	96.54	2.70
SC-FGR (%)	**92.50**	**97.50**	**97.50**	**100.00**	**100.00**	**97.50**	**2.74**

## Conclusion

This study proposes a novel SC-FGR model that consists of two parts, namely, sensing and classification of the surface EMG signal. First, wireless sensors are developed for acquiring multichannel surface EMG signals from the forearm according to the characteristics of the surface EMG signal. These sensors can provide a high-precision signal source of surface EMG for FGR. In addition, a CNN-based classification algorithm, i.e., CNN-FGR, is proposed for FGR based on the acquired surface EMG by the developed wireless sensors. The CNN-FGR is trained on a spectrum map transformed from the time-domain of surface EMG by CWT. The experimental results demonstrate that the proposed SC-FGR model achieves 97.5% recognition accuracy on eight kinds of finger gestures with five subjects, which is much higher than that of comparable models. In the future, we plan to adopt the techniques of latent factor analysis (Wu et al., 2019a, 2020, 2021a,b), cognitive computing (Wu et al., 2021c), and attention mechanism (Zheng and Chen, 2021) to simultaneously recognize the gesture and strength of the fingers based on the surface EMG of the forearm.

## Data Availability Statement

The datasets presented in this study can be found in online repositories. The name of the repository and accession number can be found below: Baidu Netdisk, https://pan.baidu.com/s/1wXT_i2kPMRALvfI17bP1YA (access code: f6wu).

## Author Contributions

JF contributed to writing—original draft, conceptualization, and methodology. SC contributed to the experiment design. LC contributed to the data curation. LY contributed to writing—review and editing. All authors contributed to the article and approved the submitted version.

## Conflict of Interest

The authors declare that the research was conducted in the absence of any commercial or financial relationships that could be construed as a potential conflict of interest.

## Publisher’s Note

All claims expressed in this article are solely those of the authors and do not necessarily represent those of their affiliated organizations, or those of the publisher, the editors and the reviewers. Any product that may be evaluated in this article, or claim that may be made by its manufacturer, is not guaranteed or endorsed by the publisher.
